# Changes in Polyphenols Contents and Antioxidant Capacities of Organically and Conventionally Cultivated Tomato* (Solanum lycopersicum L.)* Fruits during Ripening

**DOI:** 10.1155/2017/2367453

**Published:** 2017-05-25

**Authors:** Dea Anton, Ingrid Bender, Tanel Kaart, Mati Roasto, Marina Heinonen, Anne Luik, Tõnu Püssa

**Affiliations:** ^1^Department of Food Hygiene, Institute of Veterinary Medicine and Animal Sciences, Estonian University of Life Sciences, Kreutzwaldi 56/3, 51014 Tartu, Estonia; ^2^Department of Jõgeva Plant Breeding, Estonian Crop Research Institute, Aamisepa 1, 48309 Jõgeva, Estonia; ^3^Department of Animal Genetics and Breeding, Institute of Veterinary Medicine and Animal Sciences, Estonian University of Life Sciences, Kreutzwaldi 46, 51006 Tartu, Estonia; ^4^Food Chemistry, Department of Food and Environmental Sciences, University of Helsinki, P.O. Box 27, 00014 Helsinki, Finland; ^5^Department of Plant Protection, Institute of Agricultural and Environmental Sciences, Estonian University of Life Sciences, Kreutzwaldi 5D, 51014 Tartu, Estonia

## Abstract

Polyphenols of fruits and vegetables form an important part of human dietary compounds. Relatively little is known about accumulation of phenolics during fruits ripening process. The goal of this work was to study the changes in antioxidant activity and in content of 30 polyphenols during ripening of tomato fruits. Five organically and conventionally grown tomato cultivars were investigated at three different ripening stages. Phenolic compounds were extracted with methanol and extracts were analyzed by HPLC-DAD-MS/MS. During ripening, four different changing patterns were observed: (1) high level in green fruits with minimal changes; (2) continuous increase with maximum level in red-ripe fruits; (3) decrease; (4) increase and achieving maximum level at half-ripe stage. Similar change patterns were found for organic and conventional fruits. The accumulation patterns of phenolic compounds were similar in standard-type tomatoes but differed in several cases in cherry-type cultivar. Although contents of some polyphenols decreased during ripening, total phenolics and free radical scavenging activity increased in all studied cultivars and in case of both cultivation modes. The changes in content of phenolic compounds during ripening were greatly influenced by cultivars, but cultivation mode had only minor impact on dynamics in polyphenols contents in tomato fruits.

## 1. Introduction

Tomato fruit is one of the most consumed agricultural crops worldwide, cultivated in fields, greenhouses, or small home gardens. Tomato fruits have high nutritional value and additionally contain a variety of natural antioxidants, like lycopene and other carotenoids, vitamins C and E, and phenolic compounds [1]. Growing interest in polyphenols of tomato fruits is particularly connected with their antioxidant properties and possible positive health effects [2]. Fruit ripening is a biologically complex process, typically involving changes in chemical composition, pigmentation, texture, flavor, and other organoleptic characteristics [3, 4]. Tomato fruit ripening is widely studied, mainly to investigate the nutritional quality parameters, postharvesting storage conditions, and self-life extending possibilities. Although the phenylpropanoid pathway, which produces a range of secondary metabolites, including phenolic compounds, is well known [5], only limited data is available on accumulation of polyphenols, especially in organic fruits, during ripening [3, 4, 6–10].

During ripening, the tomato skin properties change [11]; fruits soften; due to chlorophyll degradation and lycopene synthesis, the green color is turning red; ethylene production increases in the respiration rate; synthesis of sugars, acids, and aroma compounds takes place [3, 12]. Gautier et al. [13] reported that the concentration of reducing sugars, carotenes, ascorbate, rutin, and caffeic acid derivatives increased, but titratable acidity, chlorophylls, and chlorogenic acid content decreased during ripening. Costa et al. [14] carried out a comparative study of three different tomato types (round, elongated, and cherry) to evaluate the mineral content in tomato fruits during postharvest ripening. They found no significant variation in Zn, P, Na, and K concentrations, but the content of Ca and Mg tended to decrease and Se content to increase. Cano et al. [15] found that lycopene accumulated throughout the ripening period; *β*-carotene content increased until breaker and pink stages and decreased afterwards; content of hydrophilic antioxidants and ascorbic acid remained practically unchanged, while the levels of aqueous phenols increased. During ripening, the total antioxidant activity increased mainly due to the increase of lipophilic antioxidants, especially lycopene [15]. Regarding polyphenols, Giovanelli et al. [1] detected different behavioral trends under different ripening conditions. Fuentes et al. [16] found that total phenolic content was higher in red tomatoes compared with green ones and in tomato peels compared with pulp and seed mucilage.

The aim of this study was to investigate the changes in polyphenols content and antioxidant capacities during ripening of organically and conventionally cultivated tomato fruits.

## 2. Materials and Methods

### 2.1. Plant Material

Plants of several tomato cultivars were grown at the Jõgeva Plant Breeding Institute, Estonia (58°44′N, 26°24′E), in two unheated plastic greenhouses with similar conditions, located next to each other. Soil at the site was classified as a soddy-podzolic sandy loam. Four standard-type tomato cultivars (cvs.) Maike, MalleF1, Valve, and Erk and a cherry-type cultivar (cv) Gartenfreude were randomly selected for the study, based on good yield and disease resistance. The first four cultivars have been bred in Estonia, at the Jõgeva Plant Breeding Institute, and were registered as new cultivars in 2003, 2004, 2003, and 1991, respectively. Cultivar Erk has the heaviest and the largest fruits (average weight 150 g) and cv Gartenfreude the lightest and the smallest ones (25 g). The organic and conventional cultivation conditions are in detail described in our previous paper [17]. Tomato fruits from both cultivation conditions, forming a sample of 0.5–1.0 kg (three replications), were collected on the same day in three ripening stages. The fruits were unripe (mature-green) at 42 days post-anthesis (DPA), half-ripe (orange) at 46 DPA, and full-ripe (red) at 48 DPA. The DPA of cherry-type cultivar were 40, 44, and 46, respectively. The fruits were sliced into segments containing flesh, skin, and seeds. Equal number of pieces of each fruit was pooled and samples were dried at 50°C using air-drier Binder FED 115 (Binder GmbH, Tuttlingen, Germany). Dry samples were ground using KnifeTec 1095 sample mill (Foss Tecator, Höganäs, Sweden).

### 2.2. Chemicals

Chlorogenic acid, caffeic acid,* p*-coumaric acid, ferulic acid, quercetin, quercetin rutinoside (rutin), naringenin, naringin, 2,2-diphenyl-1-picrylhydrazyl (DPPH), gallic acid, Folin-Ciocalteu's phenol reagent, and formic acid were purchased from Sigma-Aldrich (Steinheim, Germany). Methanol and acetonitrile were purchased from Romil (Cambridge, UK).

### 2.3. Preparation of Tomato Extracts

0.5 g of tomato powder was weighed and 5 mL 80% (v/v) aqueous methanol was added. The centrifuge tubes were placed into Biosan Multi RS60 (BioSan, Riga, Latvia) rotator for 5 hours and left overnight at room temperature. Subsequently extracts were centrifuged at 3220*g* for 10 min at 20°C using Eppendorf 5810R centrifuge (Eppendorf AG, Hamburg, Germany). The supernatants were poured into new tubes and the precipitates were reextracted. After centrifugation, two supernatants were combined and filtered using Sartorius Minisart RC4 syringe filter with pore size 0.45 *μ*m.

### 2.4. Chromatographic Analysis

The samples were analyzed using tandem liquid chromatography-diode array detection-mass-spectrometry (HPLC-DAD-ESI-MS/MS) in the negative ion mode at Agilent 1100 Series (Agilent Technologies, Palo Alto, USA) system, consisting of binary pump, vacuum degasser, autosampler, thermostated column department, diode array detector, and ion trap analyzer with electrospray ionization (ESI). For the separation of compounds, a reversed phase (RP) Zorbax 300SB-C18 column (2.1 × 150 mm; 5 *μ*m, Agilent Technologies, USA) was used. The mobile phase gradient was formed of 0.1% formic acid (solvent A) and acetonitrile (solvent B) and stepwise gradient elution program from 1% solvent B up to 28% in 1–45 minutes and in 45–55 minutes solvent B concentration was raised up to 95% and stayed for 5 minutes. During 13 minutes, the column was reequilibrated. The total run time was 73 minutes. The column flow was 0.3 mL/min and thermostat was regulated on 35°C. The injection volume of samples was 4 *μ*L. Absorption spectra were recorded online at 250, 280, 310, 325, and 360 nm. The conditions of MS/MS detection were as follows:* m/z* interval, 50–1000 amu; target mass, 400 amu; number of fragmented ions, two; maximal collection time, 100 ms; compound stability, 100%; drying gas (N_2_ from a generator) speed, 10 L min^−1^; gas temperature, 350°C; gas pressure, 30 psi; collision gas (He) pressure, 6 × 10^−6^ mbar. In MS extracted ion chromatograms (EIC), a mass window of ±0.5 Da was used. Data was collected and analyzed by Agilent 2D ChemStation Software with a ChemStation Spectral SW module.

### 2.5. Determination of Total Phenolic Content

Total phenolic content of tomato samples analyzed by HPLC-DAD-MS/MS was measured using area under curve (AUC) method described by Raal et al. [18]. The net areas under UV-Vis chromatographic curves at 280 nm between 2 and 45 min were calculated using HPLC 2D ChemStation Software. For calculations, rutin as a calibration standard was used and the results were expressed in rutin equivalents. Determination of total phenolic contents by Folin-Ciocalteu method was performed according to the procedure described in our previous paper [17].

### 2.6. Antioxidant Capacity Assay

The DPPH (2,2-diphenyl-1-picrylhydrazyl) free radical scavenging assay was performed according to the method described by Helmja et al. [19] and Fuentes et al. [16] with slight modifications. 100 *μ*L of tested extracts was mixed with 3900 *μ*L DPPH methanol solution (23.7 mg L^−1^). The absorbance was measured at 515 nm using AnalyticJena Specord200 spectrophotometer (AnalyticJena AG, Germany). The reference cuvette (blank) contained 80% (v/v) aqueous methanol. The results were expressed as the percentage of radicals scavenged after 60 minutes of reaction time.

### 2.7. Statistical Analysis

Three replicates of five tomato cultivars grown conventionally and organically were sampled at three different ripening stages forming a dataset of 90 samples in total. The study variables were MS extracted ion chromatogram (EIC) peak areas quantifying the amount of single polyphenols, total phenolic contents, and free radical scavenging capacities. To study the common change patterns among detected compounds and to compare these patterns between ripening stages, cultivars, and cultivation modes, principal component analysis was performed. As most of the concentrations of single polyphenols were not normally distributed, Wilcoxon rank-sum test was used to compare different ripening stages and cultivation modes. To take into account also potential effects of other factors, the concentrations of single polyphenols over cultivars and cultivation modes were standardized before ripening stages comparison and over cultivars and ripening stages before cultivation modes comparisons. To test the effects of ripening, cultivar, and cultivation mode and their interaction effects on total phenolic content and free radical scavenging capacity, the three-way analysis of variance was applied. The relationships between concentrations of single polyphenols, total phenolic content, and free radical scavenging capacity were studied with Pearson correlation analysis. Spearman correlation analysis was also applied, but as the statistically significant results did not change, Pearson correlation analysis was kept. In the hypothesis testing results with *p* < 0.05 were considered statistically significant, except pairwise comparison of ripening stages of single polyphenols where the approximate threshold after Bonferroni correction *p* < 0.001 was used. All statistical analyses were performed with statistical package R 3.1.1.

## 3. Results and Discussion

### 3.1. Phenolic Compounds in Tomato Fruits

A high-performance liquid chromatographic (HPLC) separation method with diode array (DAD) and mass spectrometric (MS) detection was used to determine and quantify phenolic compounds in methanol extracts of tomato fruits. Using data of retention time, mass to charge ratio (*m/z*), UV-Vis spectra, and fragmentation patterns, reported in the literature [8, 19–27] and by comparison with commercially available standards, in total 30 polyphenols were tentatively identified. UV peak areas were used for quantification of total phenols and MS peak areas for individual compounds. Tentatively identified phenolic acids belonging to the hydroxycinnamic and hydroxyphenylacetic acids and flavonoids belonging to the flavones, flavonols, flavanones, chalcones, and dihydrochalcones are presented in Table 1.

All tentatively identified compounds were found in all cultivars cultivated organically or conventionally, although some compounds were not detected in unripe green fruits.

### 3.2. Principal Component Analysis

**Figure 1:**
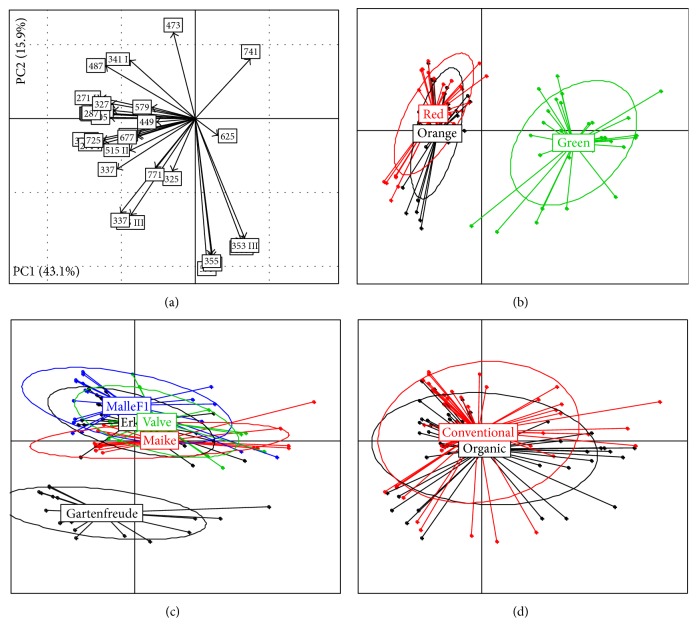
Results of PCA: (a) factor loadings of studied compounds concerning the first two principal components covering 59.0% of variability in total (numbers in figure present the* m/z* values and are explained in Table 1); ((b), (c), and (d)) factor scores grouped by ripening stage, cultivars, and cultivation mode, respectively (group label represents the group centroid).

The two first principal components describe 59.0% of the total variability of detected compounds (Figure 1). The first principal component (43.1%) mainly distinguishes unripe (green) fruits from half-ripe (orange) and red-ripe fruits, whereas the concentration of most of the compounds was higher in orange and red fruits. The second principal component (15.9%) differentiates cultivar Gartenfreude from other cultivars, indicating that there are several compounds with considerably higher or lower concentration. There is also a slight difference in values of second principal component between unripe (green) and red-ripe fruits (Figure 1(b)). The differences between half-ripe (orange) and red fruits, between other cultivars, except Gartenfreude, and between organic and conventional cultivation mode are marginal concerning the first two principal components. The third principal component (results not shown) describes 9.8% of the total variability of detected compounds and distinguishes mainly cultivars Maike and Erk. The differences between ripening stage and cultivation mode are missing. The percentage of variability described by the following principal components was less than 6% indicating that no additional common pattern exists among single polyphenols.

### 3.3. Changes during Ripening

**Figure 2:**
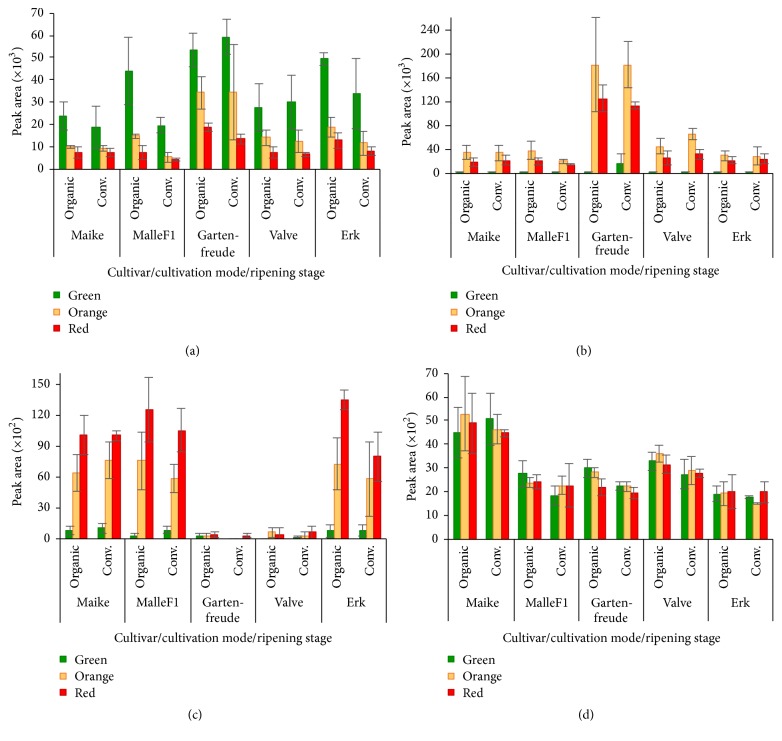
Dynamic patterns of polyphenols (mean ± standard deviation of EIC peak area) during ripening of tomato fruits. Four compounds (named in parenthesis) were selected as examples of different patterns: (a) decreasing trend (chlorogenic acid), (b) increasing and then decreasing trend (naringenin glucoside), (c) continuous increasing trend (apigenin acetylhexoside), and (d) high concentrations already in green fruits and minimal changes during ripening (kaempferol rutinoside pentoside).

Comparing the results of different ripening stages, we could draw out four basic dynamic patterns where almost all cultivars in both cultivation modes behaved in the same way. The most expressive examples of compounds with corresponding patterns are presented in Figure 2. In Figure 2(a), the highest accumulation levels were in unripe green fruits and they were continuously decreasing during ripening. In Figure 2(b), the concentrations were increasing and reaching the highest in half-ripe (orange) fruits and then slightly decreasing or remaining at the same amount. In Figure 2(c), the concentrations continuously increased and in Figure 2(d), the concentrations changed a little during ripening process.

The figures for all studied compounds are presented in Supplementary Material (see Supplementary Material available online at https://doi.org/10.1155/2017/2367453).

Assessing the compounds by chemical groups we can say that hydroxycinnamic acids with the highest number of identified compounds (16) showed different change patterns. Four compounds (both caffeic acid hexosides (*m/z* 341), homovanillic acid hexoside (*m/z* 343), and first dicaffeoylquinic acid isomer (*m/z* 515)) showed increasing trends, three compounds showed decreasing trends, and eight compounds had maximum levels at half-ripe stage. In agreement with the results of Buta and Spaulding [6],* p-*coumaric acid hexoside (*m/z* 325) had high contents already in green fruits and it remained stable during ripening.

Contents of ferulic acid hexoside (*m/z* 355) and neo- and chlorogenic acids (*m/z* 353) (Figure 2(a)) continuously decreased during ripening. Similar continuous decrease of chlorogenic acid content has been earlier reported by Buta and Spaulding [6] and Gautier et al. [13]. In contrast, the accumulation of cryptochlorogenic acid took place up to the half-ripe (in case of cherry-type tomato up to red-ripe) stage. During ripening process, the three dicaffeoylquinic acid isomers did not behave similarly, as the concentration of the first isomer was showing tendency of increase up to full-ripe stage and the other isomers increased only up to half-ripe stage and then decreased. The only identified tricaffeoylquinic acid isomer (*m/z* 677) followed the same pattern by increasing up to half-ripe stage and subsequently decreasing in all cultivars and cultivation modes. Slimestad and Verheul [2] and Moco et al. [8] reported the presence of three isomers of chlorogenic acid and di- and tricaffeoylquinic acids in all tomato tissues with increasing trend during fruit ripening. Our findings did not confirm these results, as well as the results for caffeic acid hexoside (*m/z* 341) as Moco et al. [8] found the first isomer the highest at the turning and pink stages of ripening, and we did not observe big differences in half-ripe and red-ripe fruits. The content of homovanillic acid hexoside (*m/z* 343) in all cultivars and in both cultivation modes increased continuously during ripening and reached maximum level in red fruits (depending on cultivar, 4–14 times higher than in green fruits). Dihydrocaffeic acid diglucoside (*m/z* 505) was not detected in green fruits of cv. Maike and in other cultivars was found only in low amounts. It accumulated in standard-type cultivars up to half-ripe stage, but in cherry-type cv. Gartenfreude the amounts were higher and the rise continued also in red fruits. Caffeic acid derivative (*m/z* 487) accumulated in the standard-type cultivars up to half-ripe stage; only cv. Gartenfreude was showing accumulation also in red fruits and together with cv. Valve had the lowest amounts compared to the other cultivars. Different change patterns among cultivars occurred in case of 3-(2-hydroxyphenyl)propanoic acid hexoside (*m/z* 327) and coumaroylquinic acid (*m/z* 337). As in other cultivars, the content of these compounds was the highest in half-ripe fruits, and in cultivars MalleF1 and Gartenfreude and Gartenfreude and Erk, respectively, the accumulation continued also in red fruits.

In the present study, the only compounds identified as chalcone and dihydrochalcone, naringenin chalcone (*m/z* 271) and phloretin dihexoside (*m/z* 597), respectively, followed common pattern of changes. In green fruits, naringenin chalcone was not found and phloretin dihexoside was found in minor amounts. Their levels were rising during ripening and reached maximum at half-ripe stage. Only in cv. Gartenfreude, accumulation continued also in red fruits and this cultivar had the highest quantities in all ripening stages compared with the other cultivars. We agree with the other authors [2, 22, 28] that naringenin chalcone is one of the major flavonoids in tomatoes. In our study, we had similar change pattern that was observed by Slimestad and Verheul [29] and Muir et al. [30]. In contrast to the results of Iijima et al. [22], we could not detect 50-fold higher quantities of naringenin chalcone than naringenin hexoside. This discrepancy can be explained by different drying methods. Thermal treatment used in this study might degrade more labile chalcones, and as a result we got 40-fold higher quantities of naringenin hexoside.

Assessing flavanones, we found that eriodictyol (*m/z* 287), naringenin (*m/z* 271), and their glucosides (*m/z* 449 and* m/z* 433, resp.) (Figure 2(b)) showed the tendency to accumulate up to the half-ripe stage and then slightly decrease in red-ripe fruits. Cultivar Gartenfreude showed over 10 times higher levels of these compounds compared to the other cultivars. Iijima et al. [22] did not detect eriodictyol and naringenin in their tested cultivar Micro-Tom, but in case of our cultivars only in green fruits eriodictyol was not detected and naringenin was found in very low amounts. Raffo et al. [10] found that content of naringenin increased during mid-climacteric period and after that decreased, and Iijima et al. [22] found the highest levels of naringenin 7-*O*-glucoside and eriodictyol 7-*O*-glucoside in red fruit peels. Naringin (*m/z* 579) was found in green fruits in small amounts and its concentration varied a little during ripening. In contrast to our results, Caputo et al. [31] reported increase in naringin content during ripening.

Only one flavone, apigenin acetylhexoside (*m/z* 473), was identified in our study. Its concentration continuously increased during ripening (Figure 2(c)), but in two cvs. Gartenfreude and Valve it was found in very low amounts and with minimal increase. The presence of this compound, greatly depending on the cultivar, was earlier reported by Gómez-Romero et al. [21].

Assessing accumulation changes of flavonols, only rutin hexoside (*m/z* 771), showed continuous increase reaching the highest level in red fruits. The other flavonols, rutin (*m/z* 609), quercetin dihexoside (*m/z* 625), rutin pentoside (*m/z* 741), kaempferol rutinoside (*m/z* 593), and kaempferol rutinoside pentoside (*m/z* 725) (Figure 2(d)), had maximum levels in green fruits and the concentrations stayed on the same level during ripening. Our results are in agreement with the results of studies [1, 6, 31], which found the highest levels of rutin at the earliest stage of fruit development or in mature-green fruits and observed little changes or slight decline during ripening. We also found in case of two flavonol (quercetin and kaempferol) rutinosides that two cultivars MalleF1 and Erk had different change patterns comparing cultivation modes. In organically grown fruits, these compounds decreased but in conventionally grown fruits stayed at the same level.

**Figure 3:**
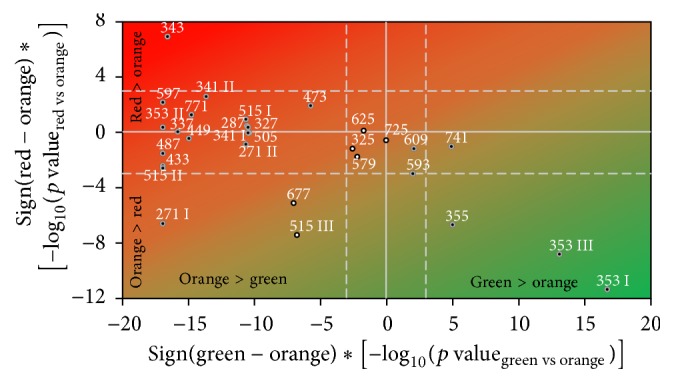
Results of Wilcoxon tests comparing orange tomatoes with green (horizontal axis) and red (vertical axis) tomatoes. The *p* values are presented in log_10_-scale (dotted lines at ±3 correspond to *p* = 0.001) multiplied by the sign of difference: in right lower corner are compounds with the highest values in green tomatoes, in left upper corner are compounds with the highest values in red tomatoes, and in left lower corner are compounds with the highest values in orange tomatoes (right upper corner corresponds to nonexisting compounds with higher values in both green and red tomatoes). Filled circles denote compounds different in green and red tomatoes (*p* < 0.001). Points' labels present compounds'* m/z* values and are explained in Table 1.

The results of Wilcoxon rank-sum test comparing different ripening stages and summarizing afore described tendencies are presented in Figure 3. There were four compounds (chlorogenic and neochlorogenic acid (*m/z* 353), ferulic acid hexoside (*m/z* 355), and rutin pentoside (*m/z* 741)) with statistically significantly higher accumulation levels in unripe green fruits compared with both half-ripe and red-ripe fruits. Additionally, the contents of two compounds, rutin (*m/z* 609) and kaempferol rutinoside (*m/z* 593), were statistically significantly higher in green fruits compared with red fruits.

There were three compounds, naringenin (*m/z* 271), tricaffeoylquinic acid (*m/z* 677), and one isomer of dicaffeoylquinic acids (*m/z* 515), with significantly higher concentration in half-ripe fruits compared with both unripe and red-ripe fruits. The concentrations of these compounds rose up to the half-ripe stage and then decreased in red-ripe stage. There was only one compound, homovanillic acid (*m/z* 343), with statistically significant higher concentration in red fruits compared with both half-ripe and unripe fruits. Furthermore, there were 18 compounds (filled circles in the left side of Figure 3) with significantly higher concentration in both red and orange fruits compared with green fruits and 16 of them showed no additional change between orange and red fruits. Four compounds,* p*-coumaric acid hexoside (*m/z* 325), naringin (*m/z* 579), quercetin dihexoside (*m/z* 625), and kaempferol rutinoside pentoside (*m/z* 725), did not show any significant changes during ripening (Figure 3).

### 3.4. Cultivars

Concentrations of some compounds were greatly dependent on the cultivar. For example, apigenin acetylhexoside (*m/z* 473) showed very low amounts in cultivars Gartenfreude and Valve (Figure 2(c)). Compared to the other cultivars, cv. Gartenfreude had the highest accumulations of 22 compounds and the lowest only in two compounds, while cv. Valve had the lowest levels of 14 compounds. In case of dihydrocaffeic acid dihexoside (*m/z* 505), eriodictyol, naringenin, and their glucosides cv. Gartenfreude had several times higher concentrations than other cultivars. Generally standard-type tomatoes all followed the same change patterns during ripening, but the cherry-type tomatoes behaved exceptionally in several cases. For example, as in other cultivars, the accumulation of the compounds increased up to half-ripe fruits, and in cultivar Gartenfreude, it continued to increase up to red-ripe fruits or vice versa (e.g., naringenin chalcone (*m/z* 271), eriodictyol hexoside (*m/z* 449), quercetin dihexoside (*m/z* 625), dihydrocaffeic acid dihexoside (*m/z* 505), and caffeic acid derivative (*m/z* 487)).

### 3.5. Cultivation Mode

Generally, cultivation mode did not influence the pattern of changes in polyphenols accumulation during ripening of tomato fruits (Figure 1(d)). With few exceptions, the studied compounds had similar change patterns in organically and conventionally grown fruits in all cultivars (Figure 2). In case of two flavonols, quercetin and kaempferol rutinosides, it was found that two cultivars MalleF1 and Erk had different change patterns comparing cultivation modes. In organically grown fruits, the content of these compounds decreased but in conventionally grown fruits stayed at the same level.

However, the statistical analysis of single compounds corrected for ripening stages and cultivars revealed that the concentration of chlorogenic acid (*m/z* 353), kaempferol rutinoside (*m/z* 593), rutin (*m/z* 609), and kaempferol rutinoside pentoside (*m/z* 725) was higher in case of organic cultivation and the content of eriodictyol (*m/z* 287) was higher in case of conventional cultivation (*p* < 0.05). But these differences in average were only in magnitude of 5–10%.

### 3.6. Total Phenolic Content and Free Radical Scavenging Capacity

**Figure 4:**
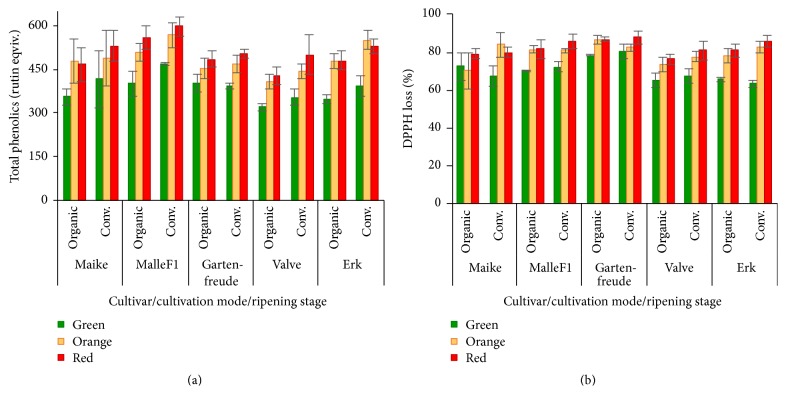
Total phenolics content by net area under curve (AUC) method (a) and free radical scavenging capacity method (b).

Total phenolic content (TPC) was determined using two different methods: chromatographic (AUC) and spectrophotometric (Folin-Ciocalteu). Between these two methods there was a strong positive statistically significant correlation (*r* = 0.60, *p* < 0.001) and also a positive correlation with free radical scavenging capacity (*r* = 0.73 and *r* = 0.68, resp., *p* < 0.001). As studied changes and associations considering total phenolic content did not depend on the determination method, in the following, the results of chromatographic method are only presented (Figure 4). The effects of cultivar, cultivation mode, and ripening were similar and statistically significant on both TPC and free radical scavenging capacity (all *p* < 0.001, except effect of cultivation mode on free radical scavenging capacity with *p* = 0.020). In average, both the TPC and free radical scavenging capacity increased during ripening in both cultivation modes and in all cultivars, being higher in case of conventional cultivation (Figure 4). The free radical scavenging capacity of tomato extracts varied slightly between tomato cultivars and the highest results were found in the fruits of cherry-type cv. Gartenfreude, while cvs. Valve and Erk showed slightly lower antioxidant activity compared to other cultivars. There were no statistically significant effects of cultivar by ripening stage, cultivation mode by ripening stage, and cultivar by cultivars mode interaction on both TPC and free radical scavenging capacity.

Caputo et al. [31] reported that the antioxidant activities of aqueous/methanol and lipophilic extracts were not correlated to each other, although in both cases deep red tomatoes had the highest antioxidant activity. Also Jesus Periago et al. [9] observed a significant increase of total phenols and antioxidant activity during ripening and found positive correlation with total phenols and flavonoid contents. Fuentes et al. [16] found also the rise of total phenols and antioxidativity during fruit ripening with the highest levels in peels, compared to pulp and mucilaginous tissue around the seeds. Slimestad and Verheul [29] found increase of total phenol contents during ripening, although content of highly antioxidative compounds such as naringenin chalcone and chlorogenic acid decreased; rutin remained quite stable and naringenin was found only in minor amounts.

## 4. Conclusions

During ripening of tomato fruits, four tendencies in changes of the levels of single polyphenols were observed: (1) high level in green fruits with minimal changes during ripening; (2) continuous increase with maximum level in red-ripe fruits; (3) decrease during ripening; (4) increase and achieving maximum level at half-ripe stage. These changes can be explained by particular roles of different polyphenols in plants. However, the literature is too scarce to compare our results with the results of other studies, especially in case of minor phenolic compounds, while chlorogenic acid, naringenin, and quercetin derivatives are more thoroughly studied.

We found that the changes in antioxidant activity during fruit ripening were similar for all cultivars. Also, our data indicated that both total phenolic content and free radical scavenging activity were rising during ripening regardless of the tested cultivar and cultivation practice used. It was also established that the cultivation mode had minor impact on changes in individual polyphenols' contents during ripening. In our study, the cherry-type cultivar in several cases behaved differently, either in the compounds' quantities or in the accumulation patterns. It shows high variability among cultivars and types of tomatoes, also unique pattern of accumulation, and degradation of every single polyphenol.

## Supplementary Material

Identification of polyphenols and dynamic patterns during ripening of tomato fruits.

## Figures and Tables

**Table 1:** List of tentatively identified phenolic acids and flavonoids.

Rt (min)	*m*/*z* [M − H]^−^	UV-Vis (nm)	Compound	MS^2^ fragment ions *m*/*z* (% intensities)	References
12.3	341	215, 290, 325	Caffeic acid hexoside I	179 (100), 135 (7), 281 (4), 251 (2), 296 (2), 223 (1), 203 (1), 161 (1)	[19, 21, 23–25]
13.3	327	220, 290, 320	3-(2-Hydroxyphenyl) propanoic acid hexoside	165 (100), 207 (3), 121 (2)	[21]
13.9	343	235, 285, 325	Homovanillic acid hexoside	181 (100), 179 (53), 135 (7), 137 (7), 251 (6), 281 (4)	[19, 21, 25]
14.2	341	235, 290, 320	Caffeic acid hexoside II	179 (100), 281 (15), 251 (13), 135 (9), 221 (2), 161 (1), 223 (1)	[21, 23–25]
14.7	325	235, 300	*p*-Coumaric acid hexoside	163 (100), 145 (80), 187 (45), 265 (17), 205 (15), 119 (9), 235 (7)	[19, 21, 23–25]
14.7	353	220, 240, 300	Chlorogenic acid^*∗*^	191 (100), 179 (2), 161 (1), 135 (1), 127 (1), 155 (1)	[19–21, 23–25]
15.6	353	205, 240, 300	Cryptochlorogenic acid	173 (100), 179 (58), 191 (30), 135 (7), 111 (1)	[19–21, 23–25]
17.0	355	240, 290	Ferulic acid hexoside	193 (100), 217 (54), 175 (34), 235 (7), 134 (5), 160 (2)	[21, 23–25]
18.2	353	220, 290, 340	Neochlorogenic acid	191 (100), 179 (3), 173 (1), 135 (1)	[20, 21, 23–25]
18.5	337	210, 235, 320	Coumaroylquinic acid	191 (100), 173 (27), 179 (6), 163 (3), 135 (1)	[20, 21, 24, 25]
19.5	771	260, 300, 350	Rutin hexoside	609 (100), 301 (8), 463 (3)	[21, 25]
21.1	473	210, 220, 270, 300	Apigenin acetylhexoside	431 (100), 288 (22), 413 (19), 269 (7), 311 (2)	[19, 21]
23.6	625	200, 280	Quercetin dihexoside	300 (100), 445 (38), 463 (16), 271 (16), 505 (13), 343 (8), 179 (4)	[21, 25, 27]
25.8	449	230, 285	Eriodictyol hexoside	287 (100), 151 (4)	[19, 22, 25]
26.0	741	200, 285	Rutin pentoside	300 (100), 609 (53), 591 (25), 271 (22), 475 (20), 255 (18), 343 (16)	[21, 23, 25]
27.6	609	210, 255, 350	Quercetin rutinoside (rutin)^*∗*^	301 (100), 271 (4), 343 (3), 255 (3), 179 (1)	[19, 21, 23–25]
28.4	725	210, 325	Kaempferol rutinoside pentoside	285 (85), 593 (65), 575 (39), 327 (27), 255 (26), 309 (12), 459 (8), 357 (4), 393 (6), 700 (6), 431 (5), 545 (5), 644 (4), 665 (4)	[8, 21]
29.3	597	235, 285	Phloretin dihexoside	477 (100), 357 (81), 387 (60), 417 (17), 459 (9), 507 (5), 579 (8), 489 (8), 315 (4), 209 (3)	[21, 25]
30.0	433	215, 285, 320	Naringenin hexoside	271 (100), 151 (2), 177 (1), 240 (1), 341 (1)	[19, 21–23, 25, 27]
30.3	515	210, 290	Dicaffeoylquinic acid I	353 (100), 173 (18), 179 (10), 191 (9), 335 (9)	[19–21, 23, 25]
30.6	515	225, 325	Dicaffeoylquinic acid II	353 (100), 191 (7), 179 (3), 173 (1)	[19–21, 23, 25]
30.7	593	235, 290, 325	Kaempferol rutinoside	285 (100), 257 (3), 229 (2), 327 (2)	[19, 21, 23, 25]
30.7	579	210, 265, 330	Naringin^*∗*^	459 (4), 271 (2), 313 (1)	
32.1	487	210, 330	Caffeic acid derivative	323 (100), 221 (12), 179 (5), 161 (3), 203 (3), 263 (2), 443 (1)	[19]
32.2	505	220, 290, 310	Dihydrocaffeic acid dihexoside	343 (89), 323 (20), 181 (31), 161 (11)	[19]
33.5	515	210, 325	Dicaffeoylquinic acid III	353 (100), 173 (13), 179 (8), 203 (10), 255 (6), 299 (6), 191 (3)	[19–21, 23]
34.9	287	210, 320	Eriodictyol	151 (100), 135 (6), 125 (3), 107 (3), 269 (3)	[19, 21, 22, 26]
40.2	271	240, 290, 335	Naringenin^*∗*^	151 (100), 177 (22), 107 (4), 125 (1)	[19, 21, 23, 26]
43.1	677	220, 280, 330	Tricaffeoylquinic acid	515 (100), 353 (18), 497 (4)	[21, 23, 26]
43.2	271	220, 250, 280, 330	Naringenin chalcone	151 (100), 177 (25), 93 (6), 165 (5), 119 (5), 107 (4)	[19, 21–23]

^*∗*^Identification confirmed by the standard compound.
