# Supplementary material for: Changes in Polyphenols Contents and Antioxidant Capacities of Organically and Conventionally Cultivated Tomato (Solanum lycopersicum L.) Fruits during Ripening
Source: Int J Anal Chem. 2017 May 25;2017:2367453. doi: 10.1155/2017/2367453 (PMC5463128; doi:10.1155/2017/2367453)
Supplement: Supplementary file 1 — Identification of polyphenols and dynamic patterns during ripening of tomato fruits. [file 2367453.f1.pdf]

Supplementary material

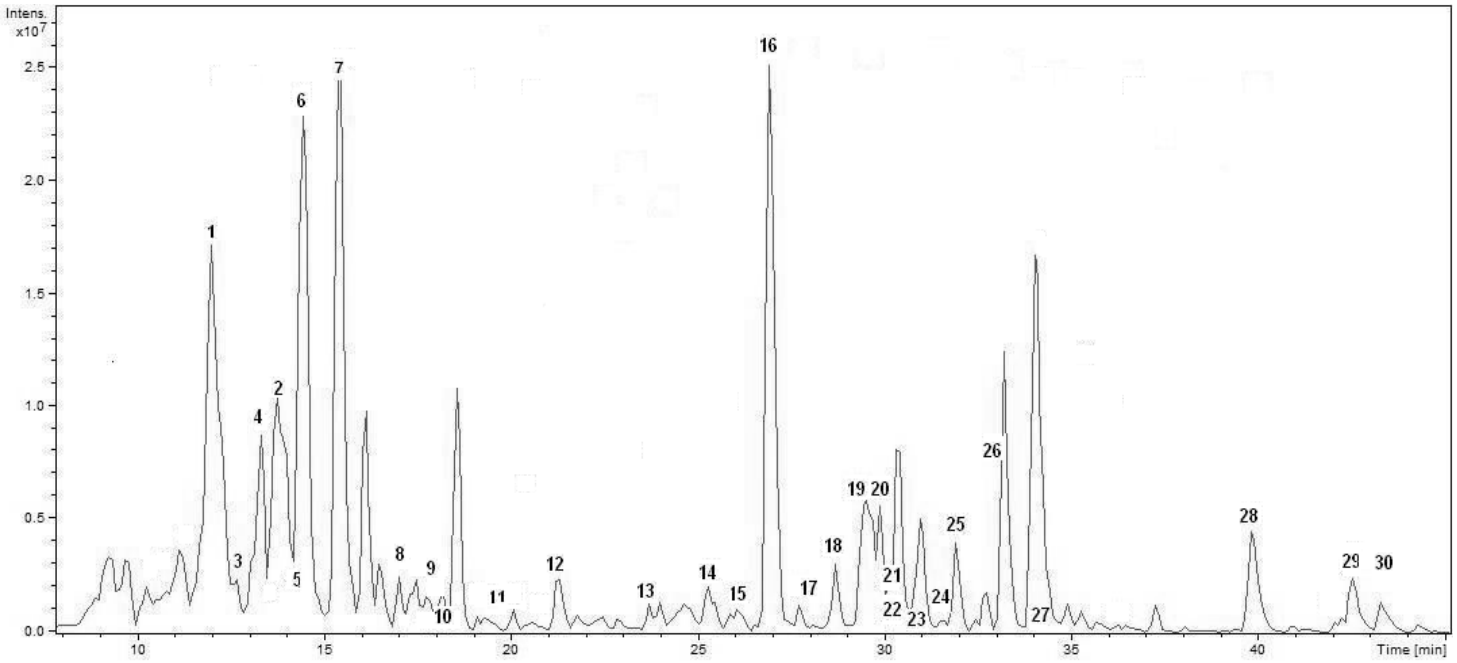

Figure1. MS-base peak chromatogram. Phenolic compounds identified by HPLC-MS/MS in tomato fruits in negative ionization mode.

1 and **2** caffeic acid hexosides (*m/z* 341), **3** 3-(2-hydroxyphenyl) propanoic acid hexoside (*m/z* 327), **4** homovanillic acid hexoside (*m/z* 343), **5** *p*-coumaric acid hexoside (*m/z* 325), **6** chlorogenic (3-caffeoylquinic) acid (*m/z* 353), **7** cryptochlorogenic (4-caffeoylquinic) acid (*m/z* 353), **8** ferulic acid hexoside (*m/z* 355), **9** neochlorogenic (5-caffeoylquinic) acid (*m/z* 353), **10** coumaroylquinic acid (*m/z* 337), **11** rutin hexoside (*m/z* 771), **12** apigenin acetylhexoside (*m/z* 473), **13** quercetin dihexoside (*m/z* 625), **14** eriodictyol hexoside (*m/z* 449), **15** rutin pentoside (*m/z* 741), **16** quercetin rutinoside (rutin) (*m/z* 609), **17** kaempferol rutinoside pentoside (*m/z* 725), **18** phloretin dihexoside (*m/z* 597), **19** naringenin hexoside (*m/z* 433) **20** dicaffeoylquinic acid I (*m/z* 515), **21** dicaffeoylquinic acid II (*m/z* 515), **22** kaempferol rutinosid (*m/z* 593), **23** naringin (*m/z* 579), **24** caffeic acid derivative (*m/z* 487), **25** dihydrocaffeic acid dihexoside (*m/z* 505), **26** dicaffeoylquinic acid III (*m/z* 515), **27** eriodictyol (*m/z* 287), **28** naringenin (*m/z* 271), **29** tricaffeoylquinic acid (*m/z* 677), **30** naringenin chalcone (*m/z* 271).

Figures 2-7. Mass spectra of selected compounds

homovanillic acid hexoside

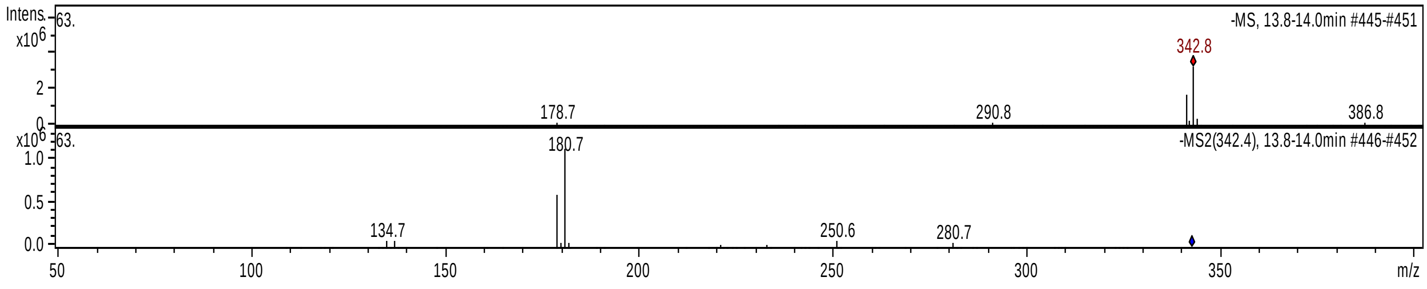

dihydrocaffeic acid dihexoside

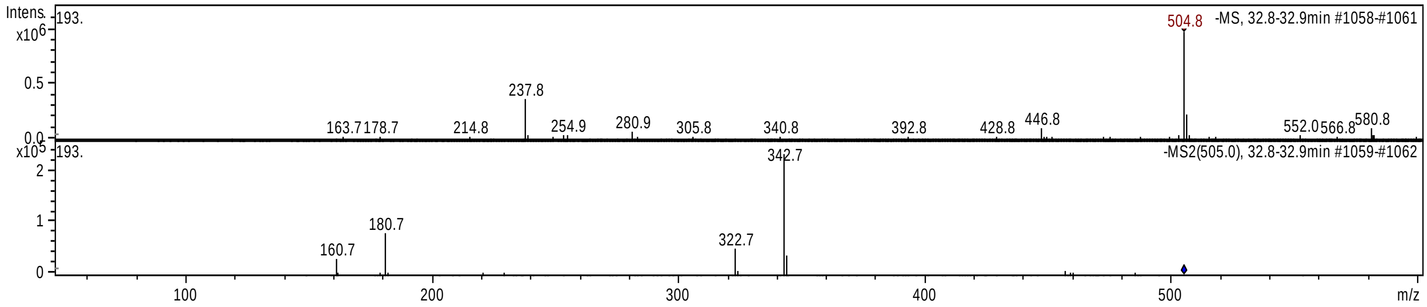

naringenin chalcone

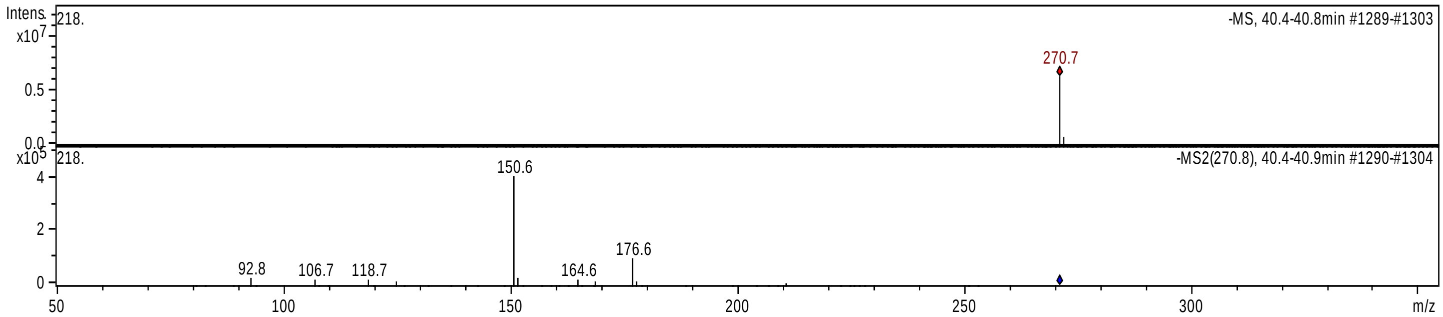

phloretin dihexoside

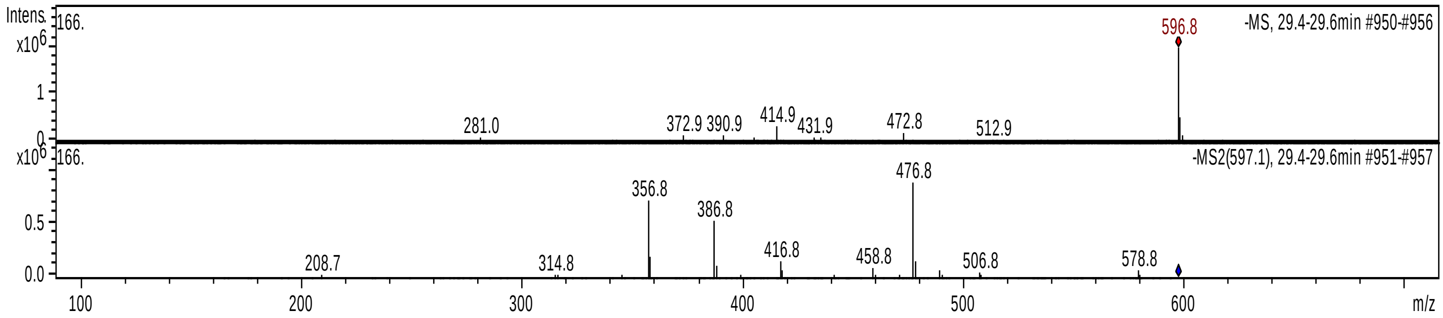

apigenin acetylhexoside *(m/z* 473)

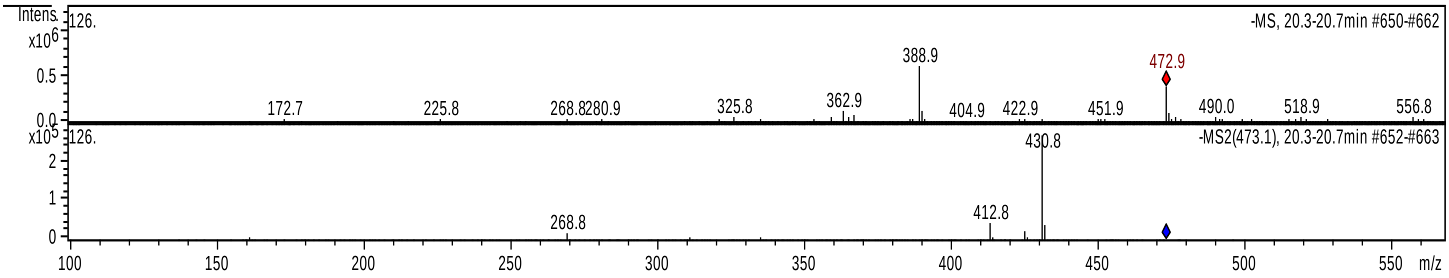

kaempferol rutinoside pentoside

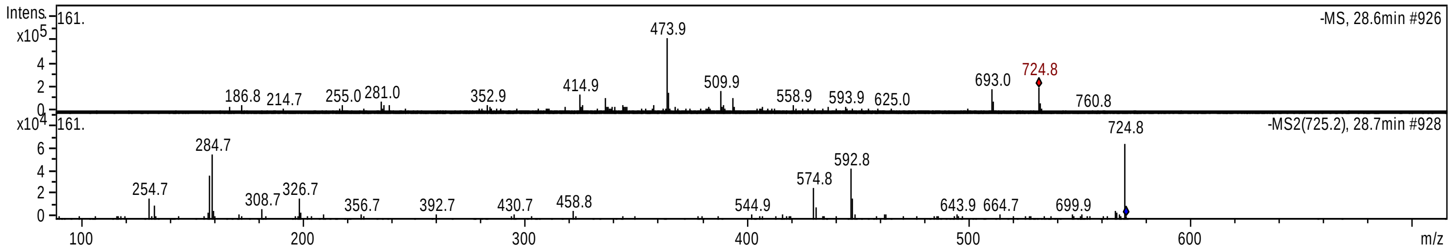

Figures 8-37: Dynamic patterns of all studied polyphenols (mean ± standard deviation of peak area) during ripening of tomato fruits.

Hydroxyphenylacetic acids

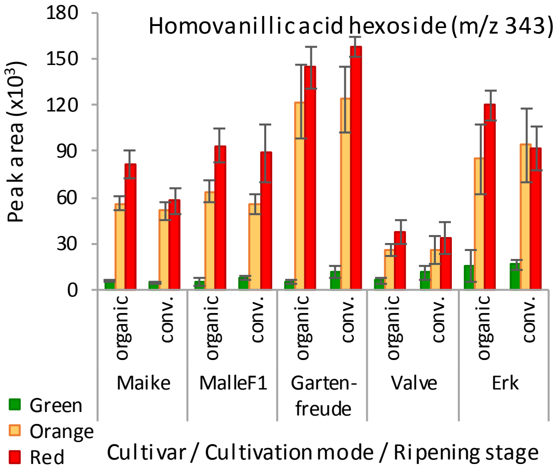

Hydroxycinnamic acids

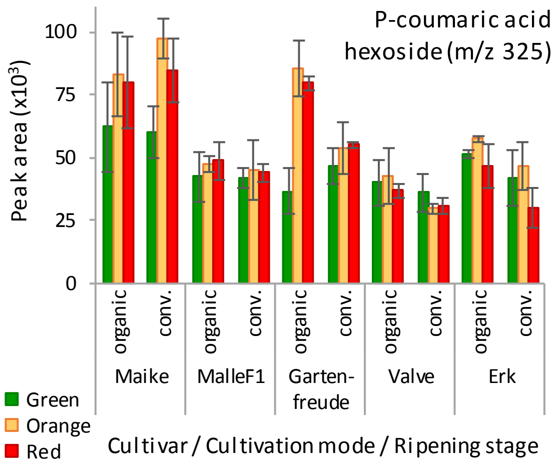

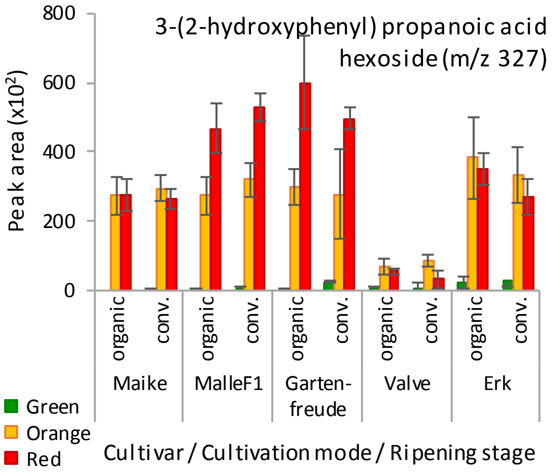

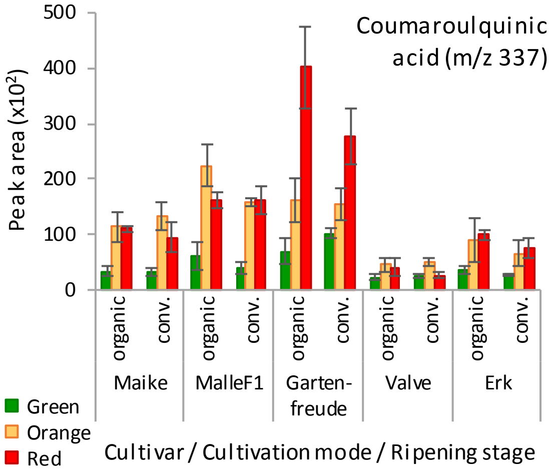

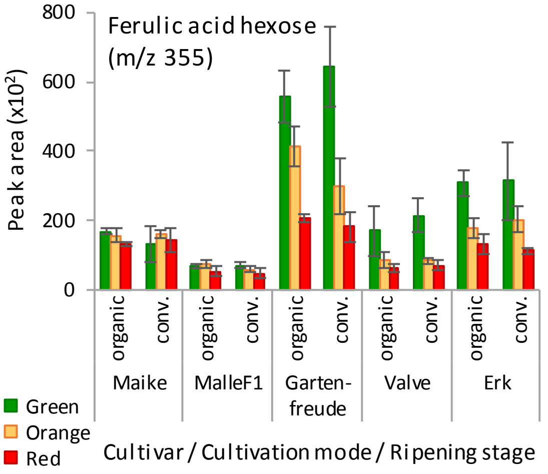

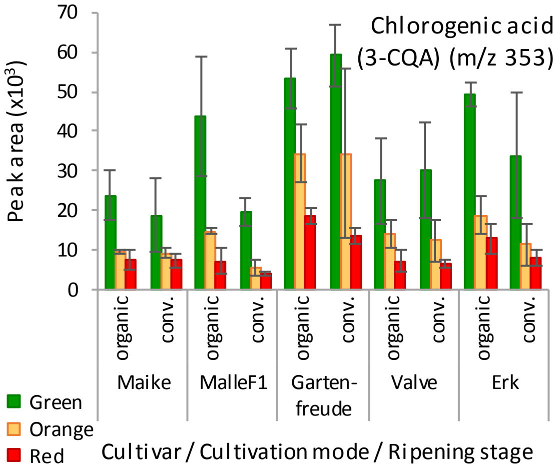
Chlorogenic acid (3-CQA) (m/z 353)
Peak area (x10^3)
0
10
20
30
40
50
60
70
organic
conv.
Maike
MalleF1
Garten-freude
Valve
Erk
Green
Orange
Red
Cultivar / Cultivation mode / Ripening stage

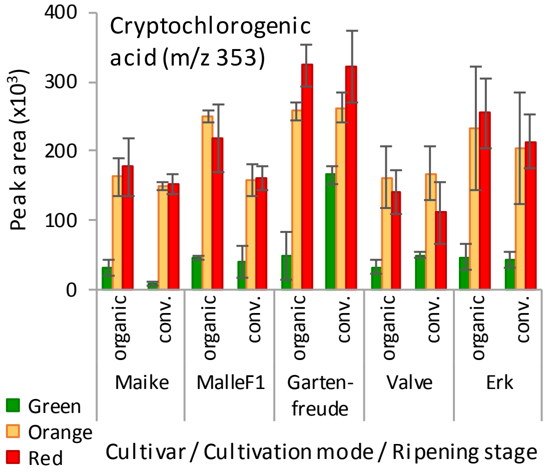
Cryptochlorogenic acid (m/z 353)
Peak area (x10^3)
0
100
200
300
400
organic
conv.
Maike
MalleF1
Garten-freude
Valve
Erk
Green
Orange
Red
Cultivar / Cultivation mode / Ripening stage

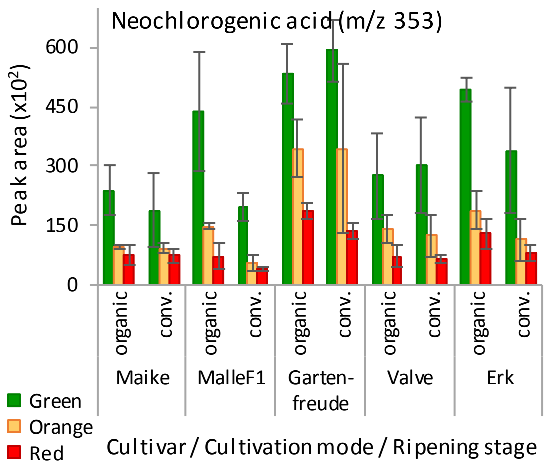
Neochlorogenic acid (m/z 353)
Peak area (x10^2)
0
150
300
450
600
organic
conv.
Maike
MalleF1
Garten-freude
Valve
Erk
Green
Orange
Red
Cultivar / Cultivation mode / Ripening stage

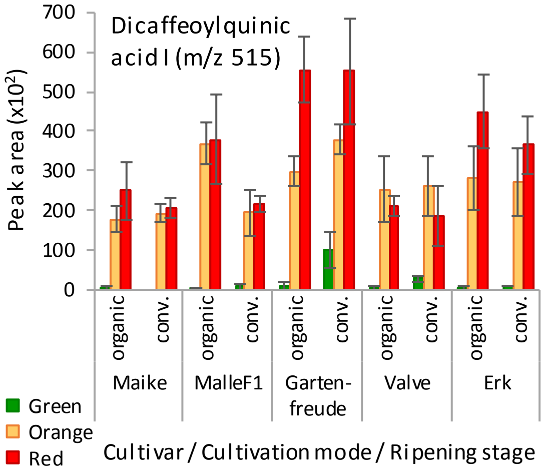
Dicaffeoylquinic acid I (m/z 515)
Peak area (x10^2)
0
100
200
300
400
500
600
700
organic
conv.
Maike
MalleF1
Garten-freude
Valve
Erk
Green
Orange
Red
Cultivar / Cultivation mode / Ripening stage

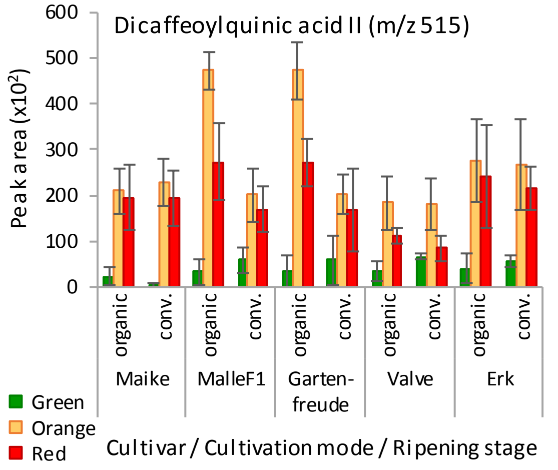
Dicaffeoylquinic acid II (m/z 515)
Peak area (x10^2)
0
100
200
300
400
500
600
organic
conv.
Maike
MalleF1
Garten-freude
Valve
Erk
Green
Orange
Red
Cultivar / Cultivation mode / Ripening stage

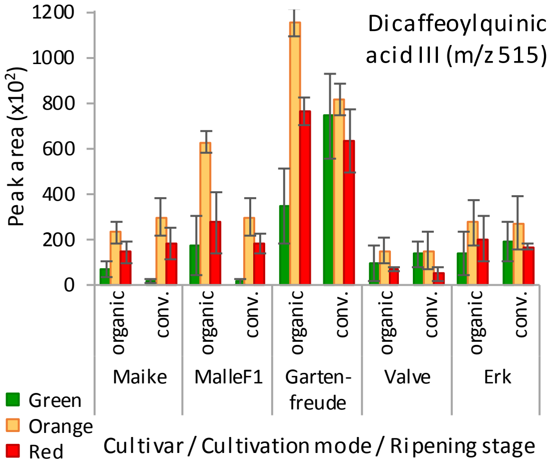
Dicaffeoylquinic acid III (m/z 515)
Peak area (x10^2)
0
200
400
600
800
1000
1200
organic
conv.
Maike
MalleF1
Garten-freude
Valve
Erk
Green
Orange
Red
Cultivar / Cultivation mode / Ripening stage

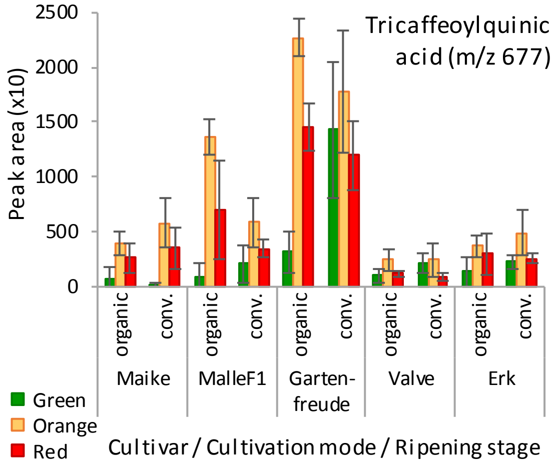
Tricaffeoylquinic acid (m/z 677)
Peak area (x10)
0
500
1000
1500
2000
2500
organic
conv.
Maike
MalleF1
Garten-freude
Valve
Erk
Green
Orange
Red
Cultivar / Cultivation mode / Ripening stage

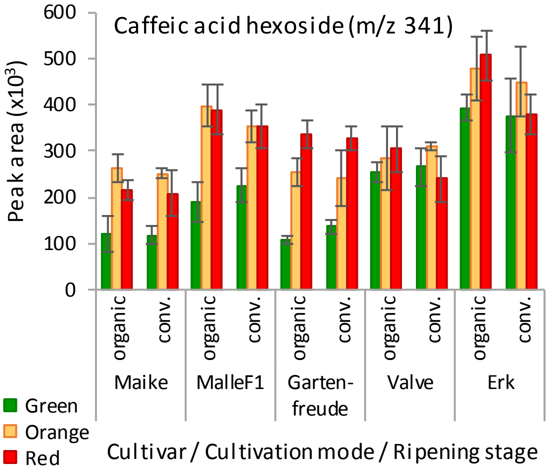
Caffeic acid hexoside (m/z 341)
Peak area (x10³)
600
500
400
300
200
100
0
organic
conv.
Maike
MalleF1
Garten-freude
Valve
Erk
Green
Orange
Red
Cultivar / Cultivation mode / Ripening stage

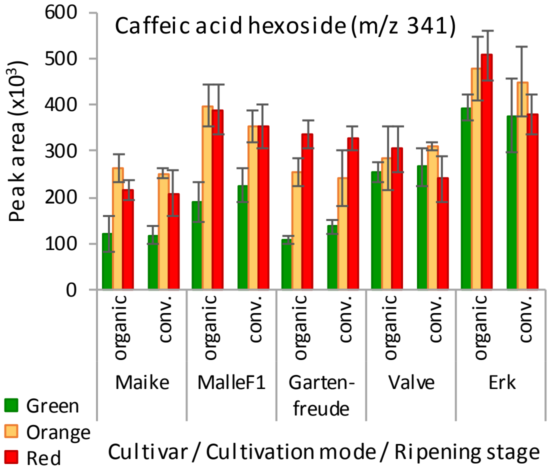
Caffeic acid hexoside (m/z 341)
Peak area (x10³)
600
500
400
300
200
100
0
organic
conv.
Maike
MalleF1
Garten-freude
Valve
Erk
Green
Orange
Red
Cultivar / Cultivation mode / Ripening stage

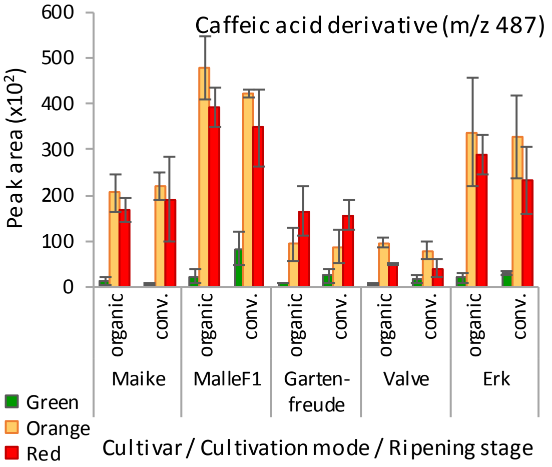
Caffeic acid derivative (m/z 487)
Peak area (x10²)
600
500
400
300
200
100
0
organic
conv.
Maike
MalleF1
Garten-freude
Valve
Erk
Green
Orange
Red
Cultivar / Cultivation mode / Ripening stage

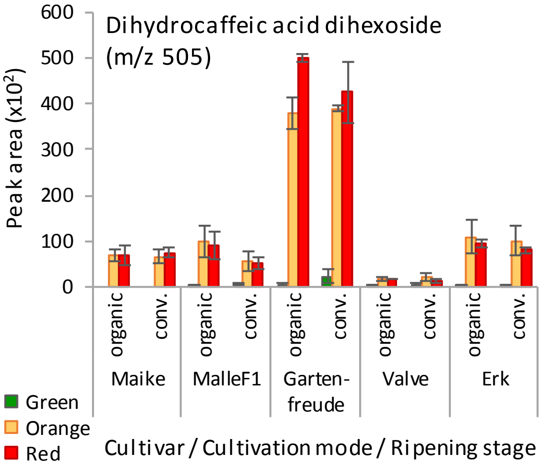
Dihydrocaffeic acid dihexoside (m/z 505)
Peak area (x10²)
600
500
400
300
200
100
0
organic
conv.
Maike
MalleF1
Garten-freude
Valve
Erk
Green
Orange
Red
Cultivar / Cultivation mode / Ripening stage

Chalcones

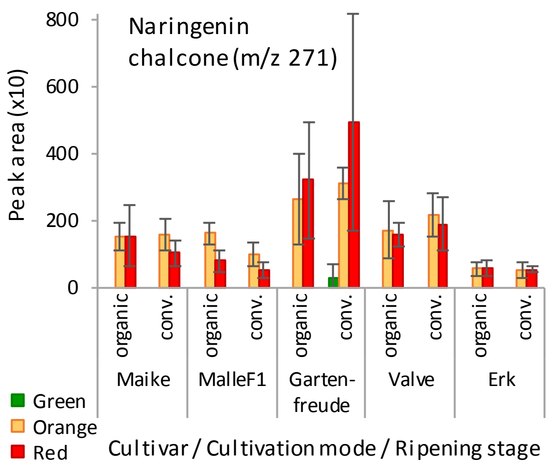

Dihydrochalcones

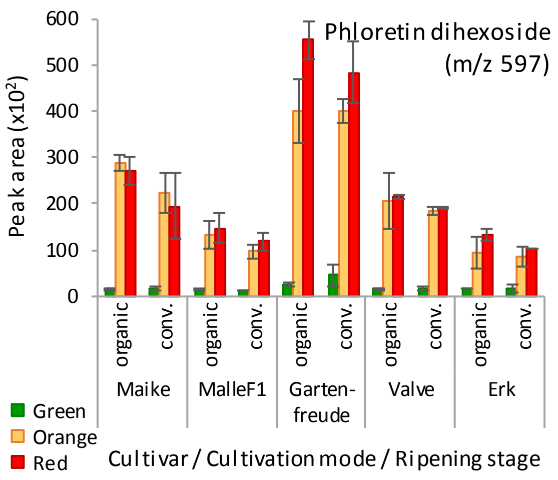

Flavones

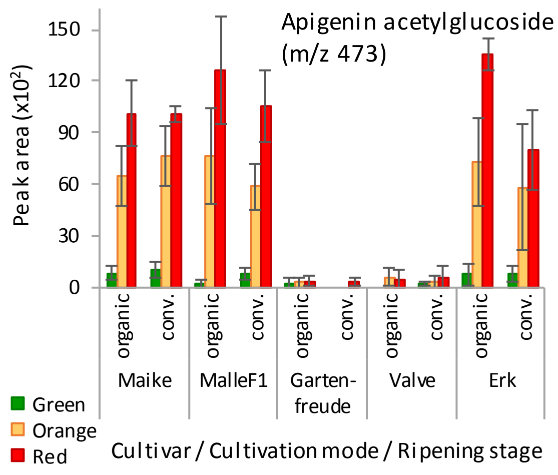

Flavonols

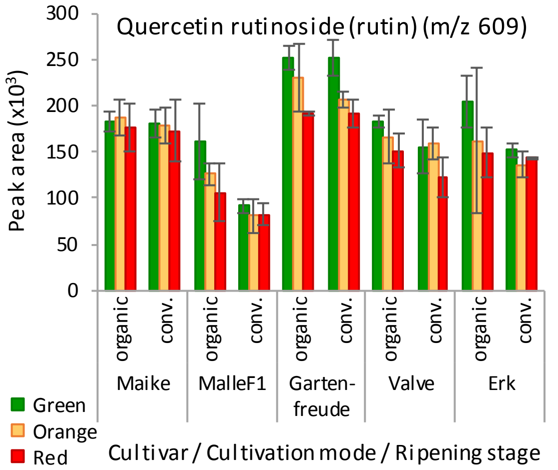
Quercetin rutinoside (rutin) (m/z 609)
Peak area (x10³)
300
250
200
150
100
50
0
organic
conv.
Maike
MalleF1
Garten-freude
Valve
Erk
Green
Orange
Red
Cultivar / Cultivation mode / Ripening stage

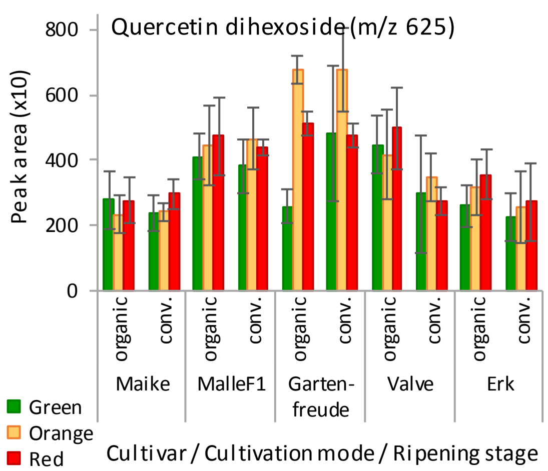
Quercetin dihexoside (m/z 625)
Peak area (x10)
800
600
400
200
0
organic
conv.
Maike
MalleF1
Garten-freude
Valve
Erk
Green
Orange
Red
Cultivar / Cultivation mode / Ripening stage

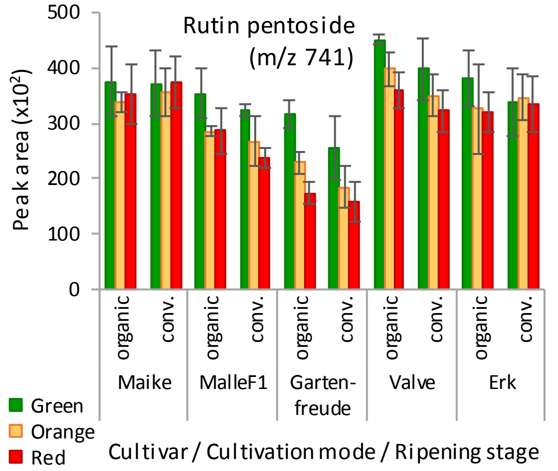
Rutin pentoside (m/z 741)
Peak area (x10²)
500
400
300
200
100
0
organic
conv.
Maike
MalleF1
Garten-freude
Valve
Erk
Green
Orange
Red
Cultivar / Cultivation mode / Ripening stage

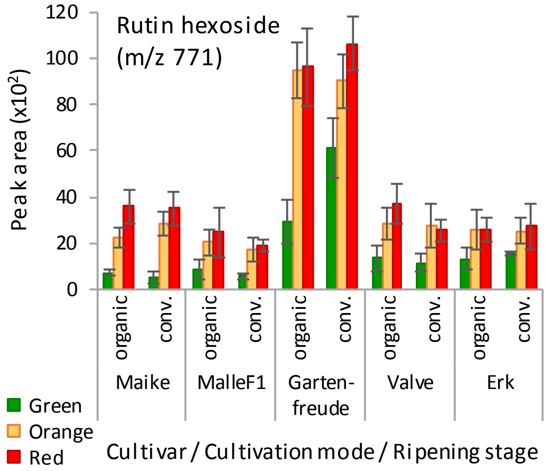
Rutin hexoside (m/z 771)
Peak area (x10²)
120
100
80
60
40
20
0
organic
conv.
Maike
MalleF1
Garten-freude
Valve
Erk
Green
Orange
Red
Cultivar / Cultivation mode / Ripening stage

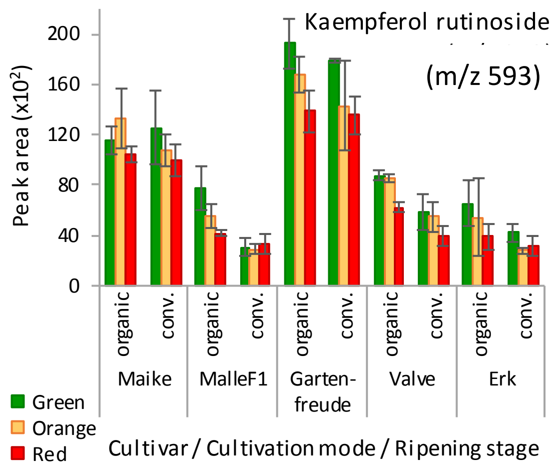
Kaempferol rutinoside (m/z 593)
Peak area (x10²)
200
160
120
80
40
0
organic
conv.
Maike
MalleF1
Garten-freude
Valve
Erk
Green
Orange
Red
Cultivar / Cultivation mode / Ripening stage

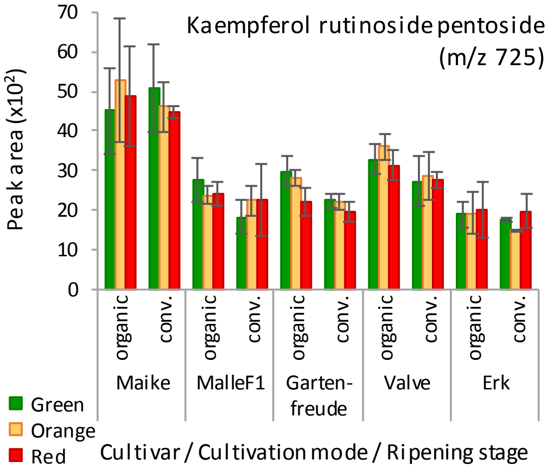
Kaempferol rutinoside pentoside (m/z 725)
Peak area (x10²)
70
60
50
40
30
20
10
0
organic
conv.
Maike
MalleF1
Garten-freude
Valve
Erk
Green
Orange
Red
Cultivar / Cultivation mode / Ripening stage

Flavanones

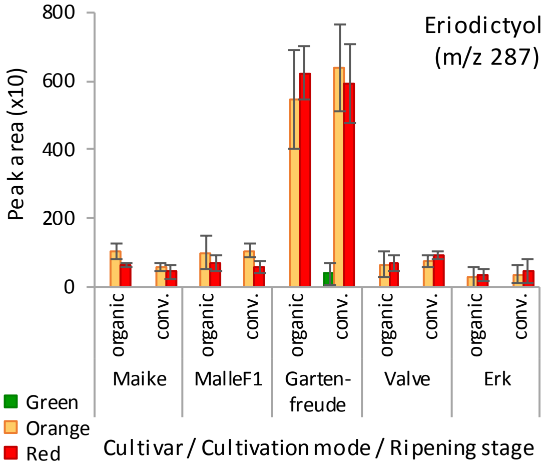
Eriodictyol
(m/z 287)
Peak area (x10)
800
600
400
200
0
organic
conv.
Maike
MalleF1
Garten-
freude
Valve
Erk
Green
Orange
Red
Cultivar / Cultivation mode / Ripening stage

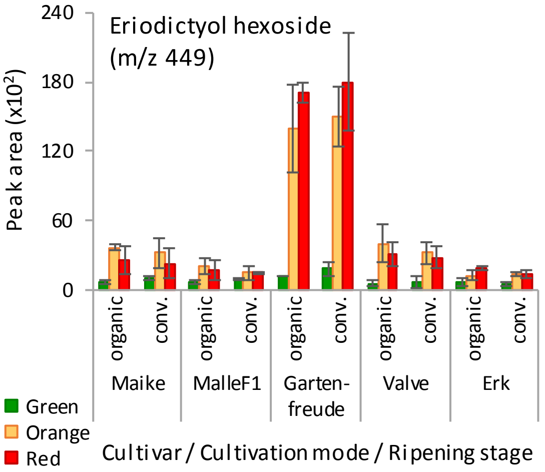
Eriodictyol hexoside
(m/z 449)
Peak area (x10²)
240
180
120
60
0
organic
conv.
Maike
MalleF1
Garten-
freude
Valve
Erk
Green
Orange
Red
Cultivar / Cultivation mode / Ripening stage

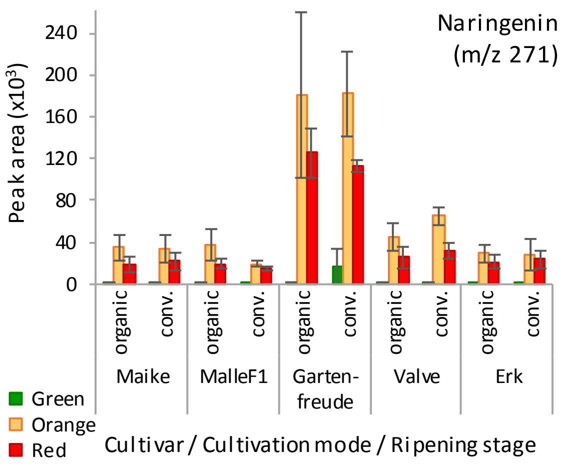
Naringenin
(m/z 271)
Peak area (x10³)
240
200
160
120
80
40
0
organic
conv.
Maike
MalleF1
Garten-
freude
Valve
Erk
Green
Orange
Red
Cultivar / Cultivation mode / Ripening stage

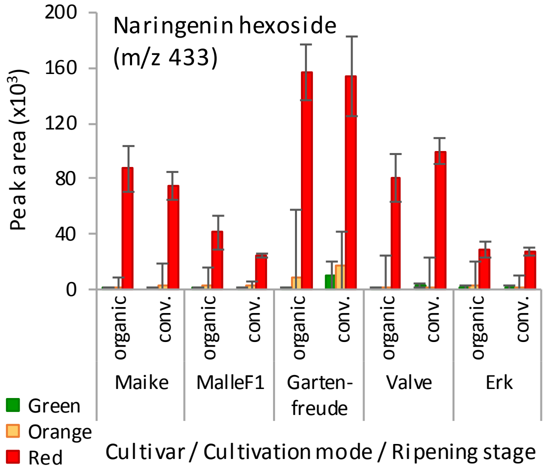
Naringenin hexoside
(m/z 433)
Peak area (x10³)
200
160
120
80
40
0
organic
conv.
Maike
MalleF1
Garten-
freude
Valve
Erk
Green
Orange
Red
Cultivar / Cultivation mode / Ripening stage

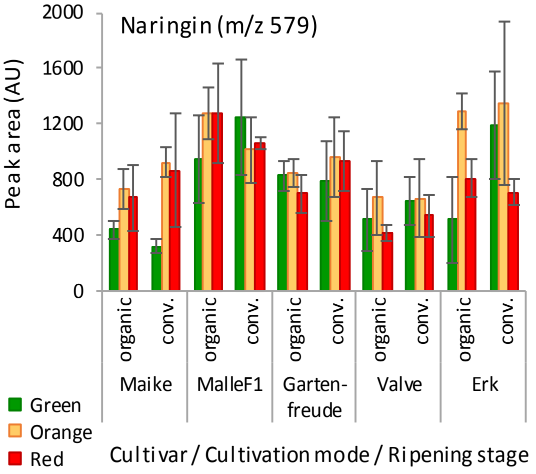
Naringin (m/z 579)
Peak area (AU)
2000
1600
1200
800
400
0
organic
conv.
Maike
MalleF1
Garten-
freude
Valve
Erk
Green
Orange
Red
Cultivar / Cultivation mode / Ripening stage
